# Historical demographic dynamics underlying local adaptation in the presence of gene flow

**DOI:** 10.1002/ece3.390

**Published:** 2012-09-27

**Authors:** Ângela M Ribeiro, Ricardo J Lopes, Rauri C K Bowie

**Affiliations:** 1 Percy FitzPatrick Institute, DST/NRF Centre of Excellence, University of Cape TownRondebosch, 7701, South Africa; 2 Museum of Vertebrate Zoology, Department of Integrative Biology, University of California3101 Valley Life Science Building, Berkeley, California, 94720, USA; 3 CIBIO Centro de Investigação em Biodiversidade e Recursos Genéticos, InBIO Laboratório Associado, Universidade do PortoCampus Agrário de Vairão, 4485-661, Vairão, Portugal

**Keywords:** Approximate Bayesian Computation, historical demography, introns, microsatellites, range limits

## Abstract

The range of a species is the result of the relative contribution of spatial tracking of environmental requirements and adaptation to ecological conditions outside the ancestral niche. The appearance of novel habitats caused by climatic oscillation can promote range expansion and accompanying demographic growth. The demographic dynamics of populations leave a signal in \ patterns. We modeled three competing scenarios pertaining to the circumstance of a range expansion by the Karoo Scrub-Robin into newly available habitat resulting from the increasing aridification of southern Africa. Genetic variation was contrasted with the theoretical expectations of a spatial range expansion, and compared with data of a putative adaptive trait. We infer that this bird likely colonized the arid zone, as a consequence of adaptive evolution in a small peripheral population, followed by an expansion with recurrent exchange of migrants with the ancestral populations.

## Introduction

Investigating the dynamics of geographical ranges of species requires the consideration of ecological and evolutionary processes acting at multiple temporal scales (Grinnell 1914; Gaston 2003; Bridle and Vines 2007; Kawecki 2008; Sexton et al. 2009). Theory pertaining to species range limits posits that peripheral populations at the margin of the species range tend to behave as sinks, unless natural selection results in local adaptation rendering them stable or even source populations (Kirkpatrick and Barton 1997). Hence, the appearance of a phenotype that allows individuals to colonize and maintain viable populations in patches outside of the ancestral area may facilitate the range expansion of the species (e.g., Myles et al. 2007; Duckworth 2008; Bocxlaer et al. 2010)

The demographic growth and selective pressures associated with range expansion into novel habitat are expected to leave a signature in genome-wide patterns of variability in contemporary populations. Population genetics theory posits that while newly advantageous variants can increase in frequency and erase previous diversity (Maynard-Smith et al. 1974; Pool and Nielsen 2007), polymorphisms that do not influence fitness are affected only by changes in the demographic trajectory of populations, unless they are linked to alleles under selection (Kimura 1956; Tajima 1989; Hudson 1990). Empirical examples have demonstrated that heterogeneous genomic divergence results from different processes (e.g., Crispo et al. 2006; Nosil et al. 2009). While migration tends to homogenize genomes, natural selection promotes the divergence of those portions of the genome that mediate adaptation to local conditions. These findings are particularly relevant to understanding the interplay between evolution and ecology in determining the range limits of species.

**Figure 1:**
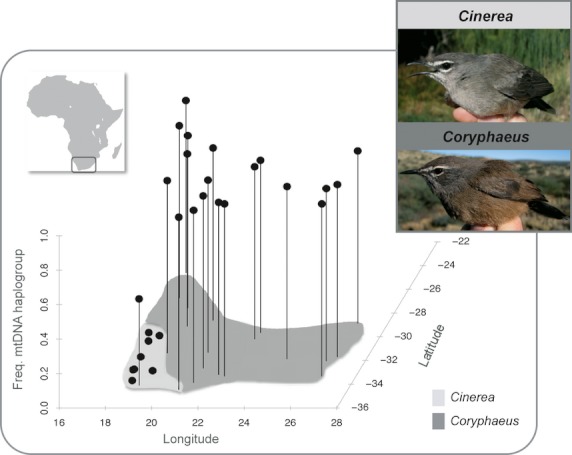
Current geographical distribution of the two subspecies *Cercotrichas c*. *cinerea* and *Cercotrichas c. coryphaeus*, underlying the spatial representation of the variation in mitochondrial DNA reported by Ribeiro et al. (2011). Inset showing the geographical location of our study system in Africa.

Since the late Miocene, the southern Africa subcontinent has experienced a trend toward increasing aridity and seasonality, with an associated shift from closed sub-tropical woodland to sparse and shrubby vegetation (Tyson 1986; Chase and Meadows 2007). The origin and establishment of the early types of shrublands and succulent vegetation (drought-tolerant flora) along the western coast and in the interior Karoo dates to the end of the Miocene (Scott et al. 1997). The regionally endemic Karoo Scrub-Robin, *Cercotrichas coryphaeus,* is a bird whose current range spans the arid and semi-arid zones of the subcontinent. This area is characterized by a west to east shift in precipitation seasonality and an aridity gradient. Plumage color differences have been used as diagnostic taxonomic traits to define two subspecies with contiguous distribution (Fig. 1): *C. c. cinerea* and *C. c. coryphaeus* (Collar 2005; Oatley 2005). The range of *Cercotrichas c. cinerea*, the form with grayish upper parts, encompasses the winter rainfall regime and associated vegetation characterized by semi-arid shrubs and dense thickets. *Cercotrichas c. coryphaeus,* the brownish colored form, occurs in the central arid area of the subcontinent, which receives most of its rain during the summer and where low shrubs dominate the sparse vegetation. A range-wide study of *C. coryphaeus* mitochondrial genomes revealed a spatial cline coincident with the climatic gradient, particularly with the west to east shift in precipitation seasonality (from winter to summer rainfall regimes) despite extensive nuclear gene flow across the whole range of the species (Ribeiro et al. 2011). These findings suggest a role for the mitochondrial genome in enabling populations to adapt to local conditions. The concordance between the ranges of the two plumage types, the environmental gradient and the mtDNA cline (Fig. 1), together with evidence about the increased aridification of the area inhabited by this species makes this a compelling system with which to explore how factors affecting population demographic dynamics, determines the evolutionary history of a species.

**Figure 2:**
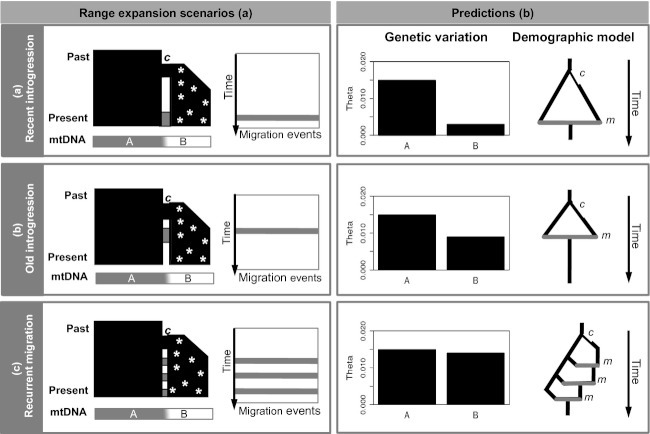
Three competing demographic scenarios of invasion of a novel habitat from a peripheral population: (a) graphical representation of the underlying demographic dynamics and current pattern of variation at mtDNA; (b) predictions of genetic variation and timing of migration events; (*c)* establishment of a small population in the new habitat (arid interior); * new adaptive trait; *m*: migration event.

We examined patterns of nuclear genetic variation in *C. coryphaeus* and compared them to population genetic predictions of the degree and spatial structure of sequence variation expected under both a demographic (Tajima 1989; Watterson 1986; Rogers and Harpending 1992) and spatial (Ray et al. 2003; Excoffier 2004; Excoffier et al. 2009) expansion to test three competing demographic scenarios (Fig. 2). In the first scenario, the appearance of a new adaptive trait in a peripheral population facilitates the establishment in the new habitat. There is a subsequent expansion of its range into the new habitat, but without exchanging migrants with the ancestral population until recent post-range expansion results in contact, and thereby extensive introgression (Fig. 2a, model A). We predict a strong signature of demographic growth, reflected as low genetic diversity and an excess of rare alleles in the colonizing population. Furthermore, the majority of coalescent events are expected to have occurred recently. In the second scenario, colonization of the novel habitat is also facilitated by a novel adaptive trait, but in this case, the new population expands and continues to exchange migrants with the ancestral population for a limited time post-colonization of the new habitat (Fig. 2a, model B). Under this model, we expect to find an excess of rare alleles in the population occupying the newly colonized habitat, moderate estimates of genetic diversity, and migration events concentrated at a given time in the past. In the third scenario, the appearance of a newly adaptive trait in a peripheral population facilitates the invasion of the new habitat and a range expansion, with the two populations continuing to exchange migrants. Under this scenario, we predict homogeneous neutral genetic variation across the entire range as a consequence of gene flow, no clear spatial pattern in the location of new and old alleles, with migration events continuing to occur through time (Fig. 2a, model C).

In all scenarios, regardless of the amount and timing of neutral gene flow, if the range and demographic expansion was facilitated by the increased fitness associated with the phenotype of a particular mitochondrial haplotype (Ribeiro et al. 2011), we expect the newly colonized population to exhibit reduced variation at the adaptive trait. Given the evidence that the Karroo Scrub-Robin is a panmictic population (Ribeiro et al. 2011), we have only considered scenarios with a single current population.

## Material and Methods

### Characterization of current climatic niche and range dynamics

The geographical distributions of the *C. coryphaeus* subspecies as defined by Oatley (2005) and Collar (2005) were used to group our point locality data into subspecies *cinerea* and *coryphaeus* (supplementary material table SI). We used a Principal Component Analysis to reduce the 10 independent bioclimatic variables (Hijmans et al. 2005) into two orthogonal axes. The PC scores were then used as dependent variables in an ANCOVA to test whether subspecies (fixed factor) occupy different climatic niches after removing the effect of latitude and longitude (covariates). All statistical analyses were performed using R (R Development Core Team 2011).

In order to assess whether the climatic requirements of *cinerea* and *coryphaeus* changed spatially during the Plio-Pleistocene climatic oscillations, and thus may have promoted a range expansion, we made use of maximum entropy models as implemented in MaxEnt v. 3.3.3. (Phillips et al. 2006a; Phillips and Dudik 2008, 2008). See supplementary material for details.

### Genetic data surveyed

#### Nuclear introns

To test among the three demographic scenarios (Fig. 2a), we used sequence data from six nuclear introns, an extension of a previously published dataset of four introns (Ribeiro et al. 2011). Sequences for Gapdh-intron11, βFib-intron5, 15691*,* and BRM-intron15 were supplemented with two further nuclear loci: TGFb2-intron 5 (*Gallus gallus* chromosome 3; Primmer et al. 2002) and 26438 (*Gallus gallus* chromosome 3; Backström et al. 2008). Details of PCR-amplifications and sequence alignment can be found in the supplementary material. The gametic phase was determined using PHASE 2.1 (Stephens et al. 2001, Stephens and Donnelly 2003), which was run twice for each locus (10^4^ main iterations and 10^3^ burn-in, -x100 option) and the highest probability haplotypes used for subsequent analyses (Harrigan et al. 2008; Garrick et al. 2010). For TGFb2, several individuals had allele pair probabilities < 80%. We designed allele-specific primers (3′-end matched one of the polymorphic sites in the template sequence) to amplify both alleles independently (see Bottema et al. 1993) and thus determine the gametic phase directly. This information was used to run PHASE (k option) once again to improve the efficiency of the statistical method to determine the gametic phase of the remaining individual.

#### Microsatellites and mtDNA

A subset (100 individuals) of a previously published dataset of 11 microsatellites (Ribeiro et al. 2011) was used to complement the demographic modeling implemented with nuclear introns, as explained under the *Demographic models section,* with the rationale that the inherent features (mutation rate) of these genetic markers would improve our characterization of the demographic trajectory of the species. The mitochondrial DNA dataset (*n* = 164; published by Ribeiro et al. (2011) was used to assess genetic variation for both the species as a whole, and for each subspecies.

### Intragenic recombination

The four-gamete test (Hudson and Kaplan 1985) was used to estimate the minimum number of recombinant events observed within each locus. As population growth is likely to mimic recombination (see supplementary material for further details), we not only used the four-gamete test as implemented in the program DnaSP (Librado and Rozas 2009) but also estimated the Φ_w_ statistic (Bruen et al. 2006) in SplitsTree v. 4.10 (Huson and Bryant 2006). The datasets were also analyzed using the single breakpoint (SPB) and genetic algorithms recombination detection (GARD) methods (Pond et al. 2006) as performed through the web interface of HyPhy (Datamonkey; Pond et al. 2005). Comparing the results from these methods provides insight with respect to the underlying demographic processes. If a signal of population growth exists in our nuclear data, we would expect the four-gamete test to recover multiple recombination events, whereas the Φw, SPB, and GARD tests should find no evidence for recombination.

### Population genetics analyses and tests of demographic change

The sequence data were used to estimate genetic diversity (*θw*; Watterson 1975) and to detect a signal of demographic expansion, as performed in DnaSP v5.10.1 (Librado and Rozas 2009). The demographic tests implemented detect departures from the Wright-Fisher model, which assumes a constant population size. We considered three tests that use distinct information contained in the sequence data: Tajima's D (Tajima 1989), *R*_2_ (Ramos-Onsins and Rozas 2002) and Fu's Fs (Fu 1997). Tajima's D and *R*_2_ are based on the allele frequency spectrum of mutations, whereas Fu's Fs is based on the haplotype distribution. For all estimates, we considered the total number of mutations rather than the number of segregating sites, because in a few instances, we observed four different nucleotides segregating. The significance of the demographic statistics was determined by comparing the empirical values with 10,000 coalescent simulations conditioned on the observed sample size and nucleotide diversity, assuming a standard neutral model with no recombination. All tests were implemented across the entire species range and also separately for each subspecies. Significantly negative Fu's Fs, positive *R*_2_, and negative Tajima's D are considered evidence for a population expansion. To infer the possible direction of the expansion, we searched for the geographical axis that maximized multilocus differentiation, estimated as the principal component scores of a covariance matrix of genetic distances among 16 sites (*N*_average_ = 5 individuals). This was conducted in R (R Development Core Team 2011) by fitting a linear model where latitude or longitude was the explanatory variable with genetic PC1 scores as the dependent variable.

### Demographic models

Using the *isolation with migration* model (Wakeley and Hey 1998; Hey and Nielsen 2007), Ribeiro et al. (2011) estimated migration rates between two populations/ecotypes of the Karoo Scrub-Robin. However, this model assumes that populations have been exchanging migrants at a constant rate since divergence. In order to reconstruct the history of colonization of the arid interior by the Karoo Scrub-Robin, we built three demographic models in which the main difference was the timing of migration events: (1) recent, (2) old, or (3) continuous. We performed Approximate Bayesian Computation (ABC) using the DIYABC package v1.0.4.41 (Cornuet et al. 2008) to infer whether the observed genetic variation is compatible with any of the three candidate demographic models. Each scenario is described as a sequence of events (Fig. 2b): the establishment of a small marginal population, period(s) of isolation, and subsequent introgression event(s). The demographic parameters used in each model and mutation rates were drawn from a range of priors reported in the supporting material (table SII). To avoid over-parameterization and assess the consistency of the selected model, analyses were implemented for the intron and microsatellite data separately. Genetic variation was summarized into six and three estimators for introns and microsatellites, respectively. We selected summary statistics that contain information about the extent of genetic polymorphism and the allele frequency spectrum. For introns, we used the number of distinct haplotypes, number of segregating sites, mean and variance of pairwise differences, Tajima's D, and the mean of the number of the rarest nucleotide at segregating sites. For microsatellites, we selected mean number of alleles across loci, mean gene diversity across loci, and mean allele size variance across loci. We simulated 5 × 10^5^ datasets per scenario and retained the 1% of the simulated data closest to the observed data to then estimate the relative posterior probability of each scenario via a direct estimates and a logistic regression.

### Demographic parameter estimates and divergence time

We used the coalescent-based *isolation with migration* model (Wakeley and Hey 1998; Hey and Nielsen 2007) to infer changes from the effective population sizes of the ancestral source population (*θ*_*a*_) to the two descendent populations (subspecies) currently occupying distinct climatic niches (*θ*_*cinerea*_, *θ*_*coryphaeus*_), as well as to estimate migration rate (*m*_*cinerea* into *coryphaeus*_ and *m*_*coryphaeus* into *cinerea*_), as implemented in IMa (Hey and Nielsen 2007). The IMa software was run multiple times to determine the appropriate prior ranges for each of the three parameters (*θ, m, and t*) and confirming proper MCMC mixing. The final run was then performed with 30 chains with a geometric heating scheme (g_1_ = 0.3 and g_2_ = 0.9). The first 1 × 10^6^ steps were discarded as the burn-in and the MCMC allowed to continue until effective sample size > 500. We used an *inheritance scalar* to adjust the parameters in the model: 0.75 – Z-linked and 1.0 – autosomal. The *Likelihood mode* was used to test whether the full model of distinct population size (*θ*_*a*_*, θ*_*1*_*, θ*_*2*_) was a significantly better fit to the data than models of constant population size (*θ*_*a*_ = *θ*_*1*_ *= θ*_*2*_). In addition to modeling population size, we also tested whether models with asymmetrical introgression (into the new habitat) were a better fit to the data than models where the directionality of migration was even, because this could provide information with respect to the directionality of expansion. The significance of the test statistics (2 × log-likelihood ratio) was assessed using a chi-squared test (Hey and Nielsen 2007). We used the point estimates of the effective size for the ancestral and descendent populations to calculate a growth ratio α_i_, whereby the growth ratio of *cinerea* α_1_ = *θ*_*cinerea*_/*θ*_*a*_*,* and *coryphaeus* α_2_ = *θ*_*coryphaeus*_/*θ*_*a*_. In order to estimate divergence time, in years, we used a species-specific generation time of 1 year (Ribeiro et al. 2012) and assumed a neutral mutation rate of 1.35 × 10^−9^ substitutions/site/year and 1.45 × 10^−9^ substitutions/site/year for the autosomal and the z-linked loci, respectively (Ellegren 2007). The geometric mean of the mutation rate for the six loci was 1.92 × 10^−6^ per substitution/locus/year.

## Results

### Range dynamics

**Figure 3:**
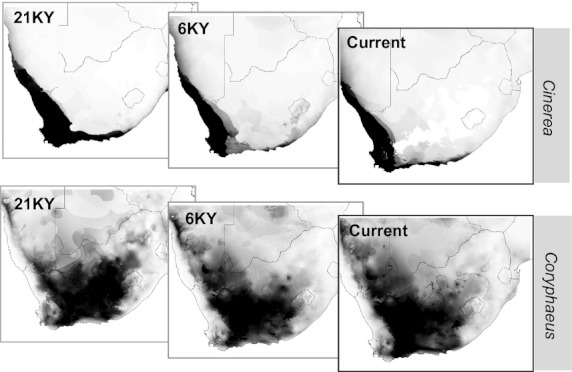
Geographical projection of the current climatic niche and predicted past range (Holocene: 6,000 ybp and Last Glacial Maximum: 21,000 ybp) of the two subspecies *cinerea* and *coryphaeus*.

After removing the possible effects of geography, the difference in the current climatic niches of *cinerea* and *coryphaeus* was highly significant (PC1_climate_: *F*_4, 53_ = 24.77, *P* < 0.001; PC2_climate_: *F*_4, 53_ = 25.94, *P* < 0.001). Distribution models, based on the current climatic niche, revealed that the Pleistocene climatic fluctuations had little effect on the paleodistribution of the subspecies (Fig. 3). The paleodistribution models further indicates that the climatic conditions currently occupied by the two subspecies have been in place since 21 000 ybp.

### Historical demography

#### Genetic variation and demographic expansion

Using the four-gamete test, we detected a few recombination events in four (Fib5, Gapdh, TGFb2, and 26438) of the six nuclear loci analyzed. The Φw test and the SPB and GARD methods did not detect any recombination events, and thus all the subsequent analyses were performed using the full sequences.

**Figure 4:**
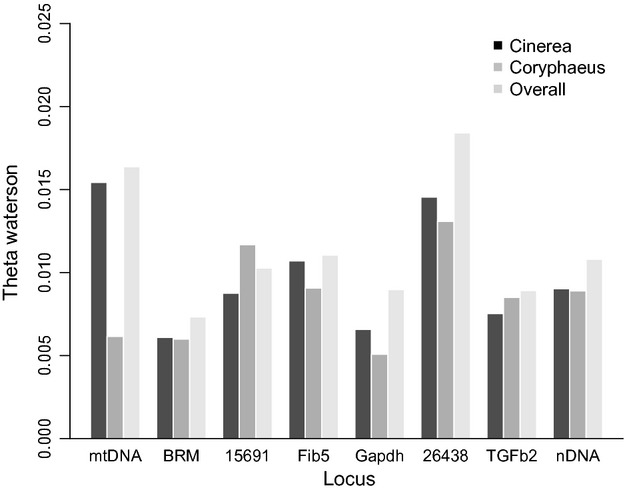
Genetic variation, *θw,* measured for mtDNA, five autosomal loci (15691, Fib5, Gapdh, 26438, TGFb2), one Z-linked locus (BRM), and overall nuclear introns for the species and within each subspecies.

Of the 1825 autosomal base pairs analyzed, 108 were polymorphic. The nuclear multilocus nucleotide diversity for the pooled samples was 0.011 (SD = 0.004). The levels of *θw* were similar between the two subspecies (Fig. 4), with the standard deviations of *θw* for *cinerea* and *coryphaeus* overlapping (0.0090 ± 0.003; 0.0089 ± 0.003). In contrast, *coryphaeus*, the subspecies that occurs in the arid interior, exhibited a significantly reduced mtDNA *θw* when compared with *cinerea*: *θw coryphaeus* (SD) = 0.0062 (0.002) versus *θw cinerea* (SD) = 0.0164 (0.002). Only two introns in *cinerea* and *coryphaeus* showed a significant skew toward an excess of rare alleles, as measured by Tajima's D. Tajimas's D values ranged from −1.578 to 0.329 in *cinerea*, and from −2.154 to −0.3319 in *coryphaeus*. Although negative in the majority of loci, coalescent simulations revealed that the observed mean values within each subspecies were not significant (Table 1). R_2_ was significant in only one locus for *cinerea*. In contrast, Fu's Fs differed significantly from expected values for every locus in *coryphaeus* and in all, but one locus in *cinerea* (supplementary material, figure S1). The shapes of the allele frequency spectra in both subspecies revealed multiple modes for the majority of the loci, that is, an excess of low-frequency alleles as well as a high proportion of intermediate frequency alleles (supplementary material, figure S2). We found no geographical trend in genetic differentiation across the whole range of the species (PC1_introns_ ∼ latitude: *r*^2^ = 0.064; PC1_introns_ ∼ longitude: *r*^2^ = 0.024) or within the range of each subspecies.

**Table 1:** Estimates of genetic diversity (θ*w*) and tests statistics of demographic change (T*D* – Tajima's D R_2_, Fu's *Fs*) across six nuclear introns. (*n*) number of individuals

	*cinerea*				*coryphaeus*				Overall			
			
Locus (*n*)	*θw*	T*D*	*R*_2_	*Fs*	*θw*	T*D*	R_2_	*Fs*	*θw*	T*D*	R_2_	*Fs*
15691 (90)	0.0088 (0.005)	**−1.578**	0.0483	**−3.760**	0.0117 (0.004)	**−2.154**	0.038	**−10.494**	0.0103 (0.004)	**−1.769**	0.0212	**−7.272**
BRM (121)	0.0061 (0.003)	**−1.530**	0.0441	**−4.430**	0.0060 (0.003)	**−1.447**	0.043	**−6.798**	0.0073 (0.003)	**−1.627**	0.029	**−10.352**
Fib5 (96)	0.0107 (0.003)	**−**0.329	0.088	**−**4.793	0.0091 (0.003)	**−**0.332	0.0083	**−8.344**	0.0111 (0.003)	**−**0.604	0.0671	**−13.670**
Gapdh (106)	0.0066 (0.003)	**−**1.152	**0.0518**	**−5.544**	0.0051 (0.002)	**−**0.770	0.064	**−2.434**	0.0089 (0.003)	**−1.512**	**0.0339**	**−19.112**
26438 (83)	0.0145 (0.006)	**−**1.413	0.0517	**−9.409**	0.0131 (0.005)	**−**1.336	0.0508	**−6.937**	0.0184 (0.006)	**−1.667**	0.0343	**−20.187**
TGFb2 (104)	0.0075 (0.003)	**−**0.679	0.0789	**−16.230**	0.0085 (0.003)	**−**1.141	0.0579	**−23.546**	0.0089 (0.003)	**−**1.075	0.0533	**−34.404**

Significant values for α = 0.05 in bold. Within brackets standard deviation.

#### Reconstructing the history of colonization and timing of migration

**Figure 5:**
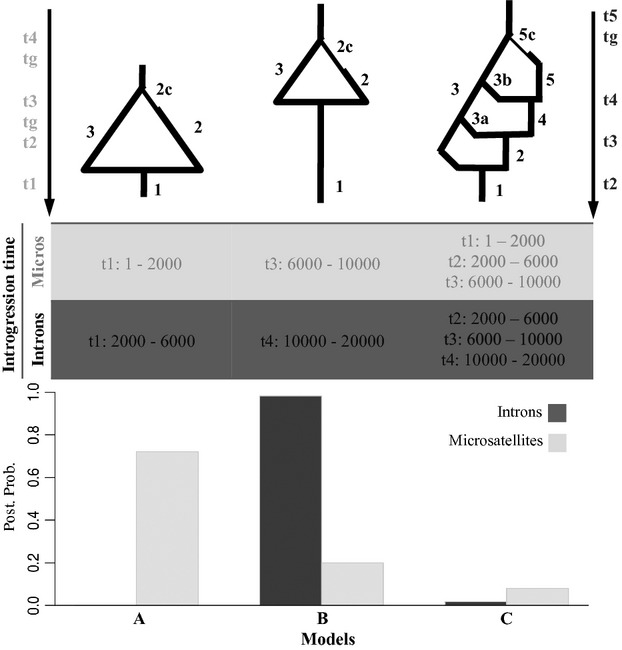
Microsatellites (light gray) and intron (dark gray) data support (posterior probability) for the three demographic scenarios (models A, B, and C) tested under the ABC framework. The main difference between models (time of introgression) is indicated in the table below the depiction of the three models. See supplementary material, table SII, for details of the priors for the size of population “1 – *i*” and timing of events “t1 – t2″.

The ABC framework used here revealed that the expansion into the arid interior (novel habitat) likely involved the establishment and demographic growth of a small marginal population that exchanged migrants at multiple times (Fig. 5). Based on the intron data, the model with highest posterior probability was model B (old introgression; *PP* = 0.9822, 95HPD: 0.9786–0.9859). In contrast, microsatellite data supported the scenario of a recent introgression (model A) with *PP* = 0.7204 (95HPD: 0.7122–0.7285). Both the direct and logistic regression methods consistently recovered the same demographic model.

#### Migration directionality, population size, and time of divergence

The simulations implemented in IMa allowed us not only to estimate migration rates and population sizes but also to compare models of divergence with directional gene flow, as well as models of constant versus distinct effective population sizes. There was a clear signal of directional migration, with migration rates from the west (*cinerea*) toward the arid interior (*coryphaeus*) being three times greater than in the opposite direction (Table 2). The size of the ancestral population was significantly smaller than the estimates of current population size obtained for *coryphaeus* (Table 2), and the effective population size of *coryphaeus* was larger than the value estimated for *cinerea,* although the 90% HPD overlapped. The growth ratio of *coryphaeus* (α_2_: 6.600) was six times larger than that of *cinerea* (α_1_: 1.311). Using likelihood ratio tests to compare nested models with the full model, we could reject all models of zero gene flow (supplementary material, table SIII), models of equal populations size, and models where the ancestral population and *coryphaeus* had the same effective size (i.e., *θ*_*1*_*, θ*_*2*_ *= θ*_*a*_; Table 3). The scaled divergence time between *cinerea* and *coryphaeus* was 0.253 (90% HPD: 0.147–0.485), which given that the mutation rates assumed corresponds to 131,144 ybp (76,205–251,425 ybp). This date corroborates the most likely demographic scenario of an old colonization of the arid interior as suggested by our ABC approach.

**Table 2:** Demographic parameters estimated under a model of *isolation with migration* using the software IMa and a dataset of six nuclear introns

Model parameters	Highest posterior probability	90% HPD
*θ ancestral*	1.3652	0.637–2.397
*θ cinerea*	1.789	0.576–5.613
*θ coryphaeus*	9.010	3.549–39.652
*m cinerea – coryphaeus*	14.050	0.050–74.550
*m coryphaeus – cinerea*	4.350	0.050–39.050
*t*	0.253	0.147–0.485

*θ,* population size; *m*, migration; *t*, time of divergence; HPD, highest posterior density.

**Table 3:** Likelihood ratio tests of nested models with equal and differential population sizes against the full model (*θ*_*1*_*, θ*_*2*_*, θ*_*a*_). *θ*_*1*_ = population size for *cinerea*. *θ*_*2*_ *=* population size for *coryphaeus*

Population size	Model	Log-likelihood (Model | data)	2LLR (df)
Equal size	*θ*_*1*_ *= θ*_*2*_ *= θ*_*a*_*, m*_*1*_*, m*_*2*_	−15.8766	**20.6294** (2)
	*θ*_*1*_ *= θ*_*2*_ *= θ*_*a*_*, m*_*1*_ *= m*_*2*_	−23.413	**35.7023** (3)
Different for *coryphaeus*	*θ*_*1*_ *= θ*_*a*_*, θ*_*2*_*, m*_*1*_*, m*_*2*_	−5.9422	0.7607 (1)
	*θ*_*1*_ *= θ*_*a*_*, θ*_*2*_*, m*_*1*_ *= m*_*2*_	−10.063	9.0024 (2)
Stable for *corypphaeus*	*θ*_*1*_*, θ*_*2*_ *= θ*_*a*_*, m*_*1*_*, m*_*2*_	−11.2608	**11.3978** (1)
	*θ*_*1*_*, θ*_*2*_ *= θ*_*a*_*, m*_*1*_ *= m*_*2*_	−15.4411	**19.7584** (2)

2LLR = 2 (log-Likelihood full model/log-Likelihood alternative model). df, degrees of freedom. Bold indicate tests that are significant for α = 0.01.

## Discussion

The interaction between the abundance and distribution of individuals, gene flow and heterogeneous environments (spatial and temporal patterns of selective pressures) determines how and when populations adapt to new conditions, and thus affect the species range dynamics. In the southern African endemic Karoo Scrub-Robin, the present occurrence data indicate that the two subspecies, *cinerea* and *coryphaeus*, have largely confined and distinct climatic niches and that their climatic requirements have been present for at least the past 21,000 years; sufficient time for local selection to have occurred. Our nuclear intron data suggest that this bird colonized the arid interior (novel habitat) during the Pleistocene (between 251,000 and 76,000 years ago), and that the effective population size for *coryphaeus,* the subspecies inhabiting the arid interior, is considerably greater than that for *cinerea*, as well as that of the ancestral population. Furthermore, by analyzing microsatellite and sequence data independently, we demonstrated that there has been effective migration between the two subspecies post the colonization of the arid interior of southern Africa. These results are consistent with a range and demographic expansion that likely was facilitated by the appearance of a newly adaptive mitochondrial variant and accompanied by neutral gene flow.

### Demographic and range expansion

Demographic dynamics during distributional range changes strongly influence genetic patterns (Excoffier and Ray 2008; McInerny et al. 2009). Spatially explicit simulations showed that when a range expansion is accompanied by demographic growth in which populations exchange large numbers of migrants (*Nm* = 100 individuals), the signature left is similar to one resulting from demographic growth alone, causing Tajima's D and Fu's Fs to be significantly negative (Ray et al. 2003). In contrast, when the number of migrants entering the population is smaller (*Nm* = 10 migrants), genealogies are characterized by coalescent events occurring both within the population and dating back to the time of expansion. As a consequence, while Tajima's D reduces its effectiveness, Fu's Fs still maintains the power to detect demographic expansions (Ray et al. 2003). The observed discrepancy between Tajima's D and R_2_, with Fu's Fs supports the scenario of range expansion with lower migration rates. However, it is important to note that ‘*Nm* = 10′ > ‘Nm = 1′ – the theoretical number of migrants required to maintain a single panmictic population (Kimura and Maruyama 1971).

The lack of a longitudinal or latitudinal trend in genetic dissimilarity across the entire range of the species or within subspecies provides further support for the scenario of range expansion followed by gene flow from the source population. Effective migration not only evens out the genetic diversity, it also spreads old alleles across the range. Furthermore, the coalescent-based estimates of migration using intron data supported the scenario of expansion into the arid zone. Gene flow was asymmetrical, with migration rates from *cinerea* into *coryphaeus* being three times larger than the estimates in the opposite direction.

The similarity in allele frequencies found at microsatellites across the range of the Karoo Scrub-Robin (Ribeiro et al. 2011) is indicative of a panmictic population. But, is it a consequence of range expansion with recurrent gene flow or the outcome of the colonization of novel habitat followed by a period of isolation and more recent contact? Although central to understanding the demographic history of populations, answering this question requires estimation of the rates and timing of migration; a task that is technically challenging as recently demonstrated via simulations by Strasburg and Rieseberg (2011). Our approach to this challenge involved the simulation of three scenarios (Fig. 2) under an ABC framework (Beaumont et al. 2002). The simulation model that generated summary statistics close to the observed values differed according to marker type: loci with slow mutation rates (introns) favored older migration events (model B), whereas more rapidly mutating microsatellite loci inferred recent gene flow (model A). Although demographic history should constrain neutral genetic variation equally, how fast mutation generates new alleles, a measure captured by our choice of summary statistics, determines the temporal sensitivity of each marker type. Thus, we interpret these seemingly contradictory results to rather be complementary, with both models providing information about the timing of migration, that is, expansion of the Karoo Scrub-Robin likely occurred with multiple instances of gene flow.

Comparing the estimates of ancestral population size with the current effective population size of both subspecies revealed a dramatic demographic expansion in the arid zone *coryphaeus* (α_2_ >> α_1_). The split between *cinerea* and *coryphaeus,* estimated from the sequence variation, dating back to the mid-Pleistocene, is consistent with the ABC model of old introgression. Although, our data violate the model assumption of invariability of population densities after divergence implemented in IMa, failing to meet such assumption causes a slightly underestimation of current population sizes, with no effect on other parameters (Strasburg and Rieseberg 2009). Thus, we contend that it has not had any meaningful effect on our inference of demographic expansion and time of divergence.

### Adaptive evolution during range expansion into novel habitats

Understanding the ecological factors maintaining or disrupting the demographic dynamics of populations at the margins of a species range has been an area of intensive research (reviewed by Sexton et al. 2009). From an ecological perspective, and without positing any adaptive change, pushing the range limits and colonizing newly available habitat depends on the niche breadth and behavioral plasticity of peripheral populations. However, a species can also expand its range by adapting to marginal habitats (Kawecki 2008): when a new trait appears and improves fitness in the peripheral sink habitat that usually characterizes the leading edge of an expansion. Recent compelling evidence has demonstrated that adaptive evolution does occur during or after the establishment of a population in a novel environment (e.g., *Homo sapiens*: Myles et al. 2007; *Bufo marinus*: Phillips et al. 2006b; *Sceloporus undulates*: Rosenblum et al. 2007).

Since the mid-Miocene, southern Africa has experienced a trend toward increasing aridification and seasonality (Cowling et al. 1999; Richardson et al. 2001). This climatic trend has altered the plant community from closed sub-tropical woodland to more open drought-tolerant biomes dominated by shrubs and succulents, with an accompanying decrease in primary productivity. These new habitats were likely characterized by new selective regimes, with respect to climate, resource availability, competitors, and predators. In the Karoo Scrub-Robin, the fact that subspecies *cinerea* and *coryphaeus* have divergent climatic niches, together with the low genetic variation found in the mtDNA of *coryphaeus*, which inhabits the geographically larger but most arid portion of the species range, suggests that expansion into a newly available habitat was likely mediated by a mitochondrial variant that enhanced individual fitness in the new climatic and nutritional niche. The congruent geographical variation in plumage coloration and mtDNA variants could simply be a by-product of a physiological response to altered climate or food resources through mitochondrial function (e.g., Das 2006). However, it could also have an adaptive function such as background matching, a potential predation avoidance mechanism given the considerable time spent by these birds on the ground while foraging; a character trait found in other ground-feeding birds inhabiting the same region, for example, *Certhilauda* Larks (Ryan et al. 1997; Dean et al. 1991).

The unprecedented availability of refined analytical methods allowed us to model the underlying historical demography of the Karoo Scrub-Robin and reveal strong support for a scenario of an adaptive range expansion with recurrent gene flow. The results obtained here give further support to the arguments articulated by Ribeiro et al. (2011) that natural selection may be maintaining the populations of this bird adapted to local conditions. However, whether the increase in frequency of a given mtDNA variant was in the first place driven by directional selection (extrinsic to the individual) or a result of niche construction (Day et al. 2003) when moving into a new habitat still remains unclear, but warrants further investigation. Our study illustrates the importance of simultaneously considering the role of ecological and evolutionary processes (Kokko & Lopéz-Sepulcre 2007): whereas, differential fitness among members of a population affect the abundance and distribution of individuals, the resulting demographic dynamics will then increase or decrease certain phenotypes in the population and thus affect evolution.

## Data accessibility

DNA sequences: Genbank accessions JN367490–JN368070, JX398334–JX398445**.** Point locality data are included in supplementary material – Table SI.

## Supporting Information

**Data S1.** Methods

**Table S1.** Point locality data used to model the geographic distribution of subspecies *cinerea* and *coryphaeus*.

**Table S2**. Parameters and priors used for each of the scenarios simulated under the ABC framework. Mutation rates were sampled from a uniform distribution: autosomal introns [1 × 10^−9^–1 × 10^−7^], Z-linked locus [1 × 10^−9^–1 × 10^–8^], and microsatellites [1 × 10^−4^−1 × 10^−3^].

**Table S3**: Likelihood ratio test of nested models of divergence in isolation (*m*_1_ *= m*_2_ *=* 0). *m*_*1*_ = migration from *cinerea* into *coryphaeus; m*_*2*_ *=* migration from *coryphaeus* into *cinera*.

**Figure S1**. Summary of the results obtained with the two most commonly used tests statistics implemented to detect departures from the constant population size model: Tajima's D and Fu's Fs. Suffix TD and Fs after locus name denote the test statistics, Tajima's D and Fu's Fs, respectively. Actual values of the summary statistic are reported in Table 1.

**Figure S2.** Allele frequency spectra for *cinerea* (red), *coryphaeus* (blue), and overall (green) for each nuclear locus are analyzed. ‘O’ and ‘E’ denote the observed variation and the variation expected under a model of stable population size, respectively.
