# Supplementary material for: Historical demographic dynamics underlying local adaptation in the presence of gene flow
Source: Ecol Evol. 2012 Sep 27;2(11):2710–21. doi: 10.1002/ece3.390 (PMC3501624; doi:10.1002/ece3.390)
Supplement: Supplementary file 1 [file ece30002-2710-SD1.pdf]

SUPPLEMENTARY MATERIAL for "Historical demographic dynamics underlying local adaptation in the presence of gene flow" by Ribeiro and colleagues.

The PDF includes:

1. Methods
2. Tables: SI and SII
3. Figures: S1 and S2

1. Methods

Characterization of current climatic niche and range dynamics

We combined presence-only data (46 points for *coryphaeus* and 17 points for *cinerea*) with climatic layers to first model the current distribution of the Karoo Scrub-Robin and the two subspecies at a resolution of 1 km^2 using ten independent bioclimatic variables (Pearson's correlation coefficient $r < 0.70$) produced by Hijmans et al. (2005). The accuracy of the models was assessed by partitioning the occurrence data into training (80%) and testing (20%) subsets, as well as by estimating the "area under the receiver operating curve" – AUC. Point-locality data used in these models were obtained primarily from our field expeditions and supplemented with a few records from colleagues (Peter Nupen, Dawie de Swardt and Gordon Schultz, personal communication). Although museum specimens were available, the geographic coordinates were not detailed and thus, we chose to have fewer, but more accurate points. The potential paleo-geographical range of the species and subspecies were inferred by projecting the current model into two time periods, representing the extreme climatic conditions during the Quaternary: the Last Glacial Maximum (21 000 years before present - ybp) and Holocene Optimum (6 000 ybp). Data from the Paleoclimatic Modelling Intercomparison Project ECHAM3, downscaled at the 30 s (1 km^2) resolution were used to reconstruct the past climate surfaces.

Genetic data surveyed - nuclear introns

PCR-amplifications were performed in a total volume of 10 µl with 10-20 ng of genomic DNA, GeneAmp 10x PCR Gold Buffer, 2.0-2.5 mM $MgCl_2$, 0.3 mM of each dNTP, primer concentrations of 0.15 mM and 0.5 units of Taq polymerase (Roche). The thermocycling profile comprised an initial denaturizing step at 95°C for 3 min followed by 35 cycles at 95°C

for 30 seconds, a locus-specific annealing temperature of 55°C-60°C for 30 seconds, and 72°C for 30 seconds, with a final 7 min extension step at 72°C. PCR products were cycle sequenced in both forward and reverse directions using the ABI BigDye Terminator Kit v3.1 (Applied Biosystems) and then analyzed on an AB3730 automated sequencer. Sequences were then edited and aligned using CodonCode Aligner v3.5.2 (CodonCode Corporation) and Geneious Pro v5.0 (Biomatters Ltd). We validated several rare alleles (low frequency variants) by repeating the amplification and sequencing protocols. Multiple length-polymorphisms were found at the locus *26438*. We thus truncated the sequences at the first indel.

Intragenic recombination

Under the infinite sites model (ISM; Kimura 1969) the occurrence of recombination between two polymorphic sites at a given locus will originate four different haplotypes: two recombinants and two parental (Hudson and Kaplan 1985). This expectation is used in the four-gamete test to estimate the minimum number of recombinant events observed within each locus. However, during population growth the increasing number of mitotic events, and hence mutations, is likely to create new haplotypes that mimic recombination. Under such a demographic scenario the ISM is violated and recombination events can be overestimated. Therefore, besides using the four-gamete test to detect intragenic recombination events, we also used the Φw statistic; the latter test (Φw) measures the genealogical similarity between closely linked sites and thus discerns whether it is due to recurrent mutation or recombination.

2. Tables

Table SI. Point locality data used to model the geographic distribution of subspecies. *cinerea* and *coryphaeus*.

Locality	Country	Latitude	Longitude
Aliwal North	South Africa	-31.6978	26.6842
Anysberg NR	South Africa	-33.4632	20.5886
Anysberg1	South Africa	-33.4590	20.5907
Anysberg2	South Africa	-33.4615	20.5925
Anysberg3	South Africa	-33.4649	20.5898
Augrabies SANP	South Africa	-28.6180	20.3506
Augrabies SANP17	South Africa	-28.5934	20.3343
Beaufort West	South Africa	-32.4000	22.5667
Beaverlac	South Africa	-32.8681	19.0832
Bontebok NP	South Africa	-34.0075	20.5197
Bulletrap	South Africa	-29.8786	18.4494
DeHoop	South Africa	-34.4179	20.6888
DeRust	South Africa	-33.3201	22.3233
Dorinckloof	South Africa	-33.8667	21.1333
Dwarskersbos	South Africa	-32.7142	18.2416
Ficksburg	South Africa	-28.8664	27.8649
Fraserburg	South Africa	-31.8550	20.9831
Garies	South Africa	-30.5363	18.0345
Garies_Wolfhok Farm	South Africa	-30.3734	18.0992
Grahamstown	South Africa	-33.2704	26.4884
Helderberg	South Africa	-34.0562	18.8723
Kalkwal1	South Africa	-28.8336	26.6843
Kalkwal2	South Africa	-28.8341	26.6840
Karasburg1	Namibia	-27.5528	18.9574
Karasburg2	Namibia	-27.5621	18.9598
Koeberg	South Africa	-33.6793	18.4347
Kraaifontein	South Africa	-31.3397	19.1072
Kuruman	South Africa	-27.4853	23.4431
Loeiesfontein	South Africa	-31.0711	19.2233
Mariental	Namibia	-24.5919	17.9406
Merweville_NovaVita Farm	South Africa	-32.6406	21.1833
Nieu Bethesda	South Africa	-31.8500	24.5500
Nieuvoldtville_GlenLyon	South Africa	-31.4075	19.1483
Nieuwouldtville_ESwartberg	South Africa	-31.3742	19.2117
North Keetmanshoop	Namibia	-26.4098	18.1771
Noupoort	South Africa	-31.2333	24.9500
Papendorp	South Africa	-31.7053	18.1978
Poffadder1	South Africa	-29.1191	19.3767
Poffadder2	South Africa	-29.0955	19.3788
Potfontein1	South Africa	-30.2000	22.6326
Potfontein2	South Africa	-30.1858	22.7105

Prieska_camp1	South Africa	-29.6618	22.7597
Prieska_camp2	South Africa	-29.6500	22.7500
Prieska_farm1	South Africa	-29.6529	22.7414
Prieska_farm2	South Africa	-29.6586	22.7626
Prieska_farm3	South Africa	-29.6486	22.7564
Prieska_farm4	South Africa	-29.6921	22.7468
Prince Albert	South Africa	-33.1833	22.1000
R361 Van Zyksvlei to	South Africa	-30.6949	22.0384
R383 Postmasburg to Prieska	South Africa	-28.9834	22.6812
Reit and Modder confluence	South Africa	-28.9850	26.4059
Rouxville	South Africa	-30.4142	26.8342
South Keetmanshoop1	Namibia	-26.7375	18.3292
South Keetmanshoop2	Namibia	-26.6958	18.3208
Springbock	South Africa	-29.6634	17.8953
Strydenburg	South Africa	-30.6212	24.5845
Tarkastad	South Africa	-32.0394	26.2933
Three Sisters	South Africa	-31.8883	23.0839
Tygberg	South Africa	-33.8806	18.5997
Van Wyksvlei Dam	South Africa	-30.3785	21.8118
Velddrift	South Africa	-32.7564	18.1701
West of Carnavon_Farm	South Africa	-31.1024	21.7210
Williston	South Africa	-31.3272	20.9831

Table SII. Parameters and priors used for each of the scenarios simulated under the ABC framework. Mutation rates were sampled from a uniform distribution: autosomal introns [1 x10^{-9} - 1 x10^{-7}], Z-linked locus [1 x10^{-9} - 1 x10^{-8}] and microsatellites [1 x10^{-4} - 1 x10^{-3}].

Scenario	Parameter	Description	Priors Range
**Colonization with recent introgression (model A)**	N1, N2, N3	population size	[100 - 100 000]
	N2c	marginal population	[10 – 500]
	t1 / t2	time introgression	[1 - 6 000] /]2 000 – 6 000]
	tc	time of establishment	[1- 500]
	t2 / t4	time of divergence	]2 000 – 6 000] /]10 000 – 20 000]
	ra1	introgression rate	[0.001 – 0.999]
**Colonization with old introgression (model B)**	N1, N2, N3	population size	[10 - 100 000]
	N2c	marginal population	[10 – 500]
	t3 / t4	time introgression	[1 - 6 000]
	tc	time of establishment	[1- 500]
	t4/ t5	time of divergence	]10 000 – 20 000] /]20 000 – 300 000]
	ra1	introgression rate	[0.001 – 0.999]
**Colonization with recurrent migration (model C)**	N1, N2, N3, N3a, N4, N3b, N5	population size	[10 - 100 000]
	N5c	marginal population	[10 – 500]
	tc	time of establishment	[1- 500]
	t1 / t2	time introgression	[1 - 2 000] /]2 000 – 6 000]
	t2 / t3	time introgression	]2 000 – 6 000] /]6 000 – 10 000]
	t3 / t4	time introgression	]6 000 – 10 000] / [10 000 – 20 000]
	t4 / t5	time of divergence	]10 000 – 20 000] /]20 000 – 300 000]
	ra1, ra2, ra3	introgression rate	[0.001 – 0.999]

Table SIII: Likelihood ratio test of nested models of divergence in isolation (m_1 = m_2 =0). m_1 = migration from *cinerea* into *coryphaeus;* m_2 = migration from *coryphaeus* into *cinera.*

	Model	logLikelihood (Model | data)	2LLR (df)
Isolation	$\theta_1 = \theta_2 = \theta_a$, $m_1 = m_2$	-460.517	**909.9103 (4)**
	θ_1 , $\theta_2 = \theta_a$, $m_1 = m_2 = 0$	-460.517	**909.9103 (3)**
	$\theta_1 = \theta_2$, θ_a , $m_1 = m_2 = 0$	-460.517	**909.9103 (3)**
	$\theta_1 = \theta_a$, θ_2 , $m_1 = m_2 = 0$	-460.517	**909.9103 (3)**
	θ_1 , θ_2 , θ_a , $m_1 = m_2 = 0$	-460.517	**909.9103 (2)**

df: degrees of freedom. Bold indicate tested that are significant for α = 0.01.

3. Figures

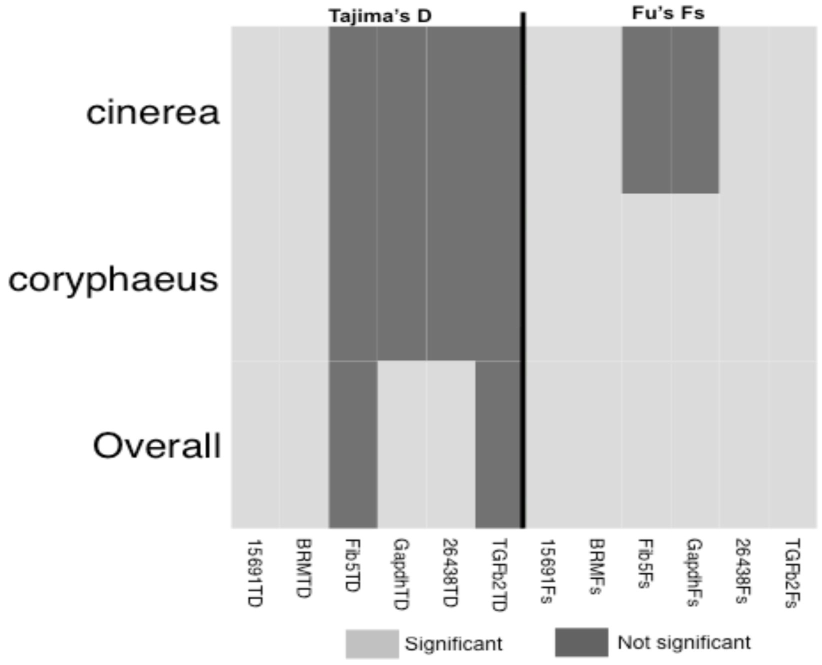

Figure S1. Summary of the results obtained with the two most commonly used tests statistics implemented to detect departures from the constant population size model: Tajima's D and Fu's Fs. Suffix TD and Fs after locus name denote the test statistics, Tajima's D and Fu's Fs, respectively. Actual values of the summary statistic are reported in Table I.

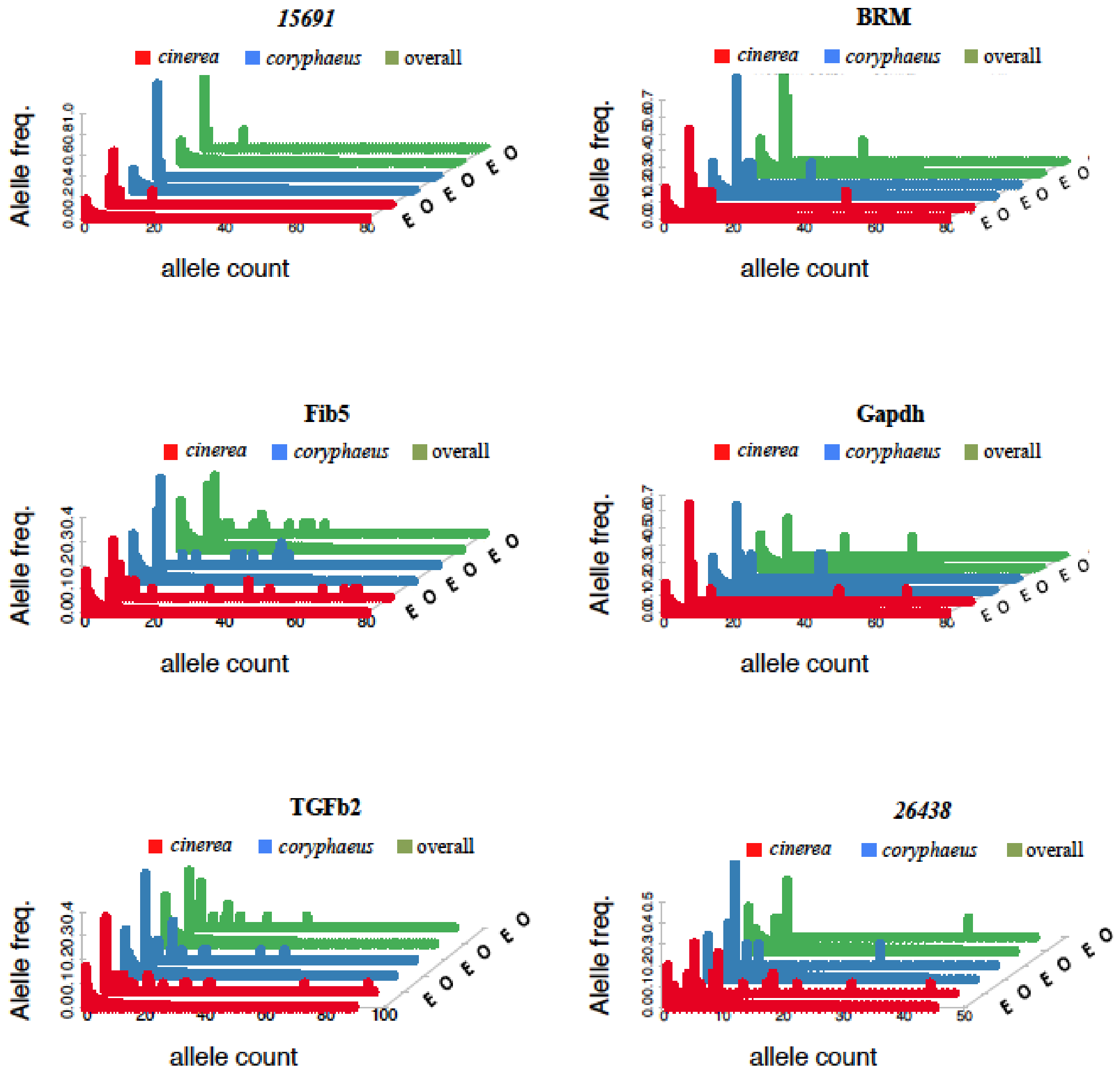

Figure S2. Allele frequency spectra for *cinerea* (red), *coryphaeus* (blue) and overall (green) for each nuclear locus analysed. 'O' and 'E' denote the observed variation and the variation expected under a model of stable population size, respectively.
