# Supplementary figures and images for: Historical demographic dynamics underlying local adaptation in the presence of gene flow
Source: Ecol Evol. 2012 Sep 27;2(11):2710–21. doi: 10.1002/ece3.390 (PMC3501624; doi:10.1002/ece3.390)

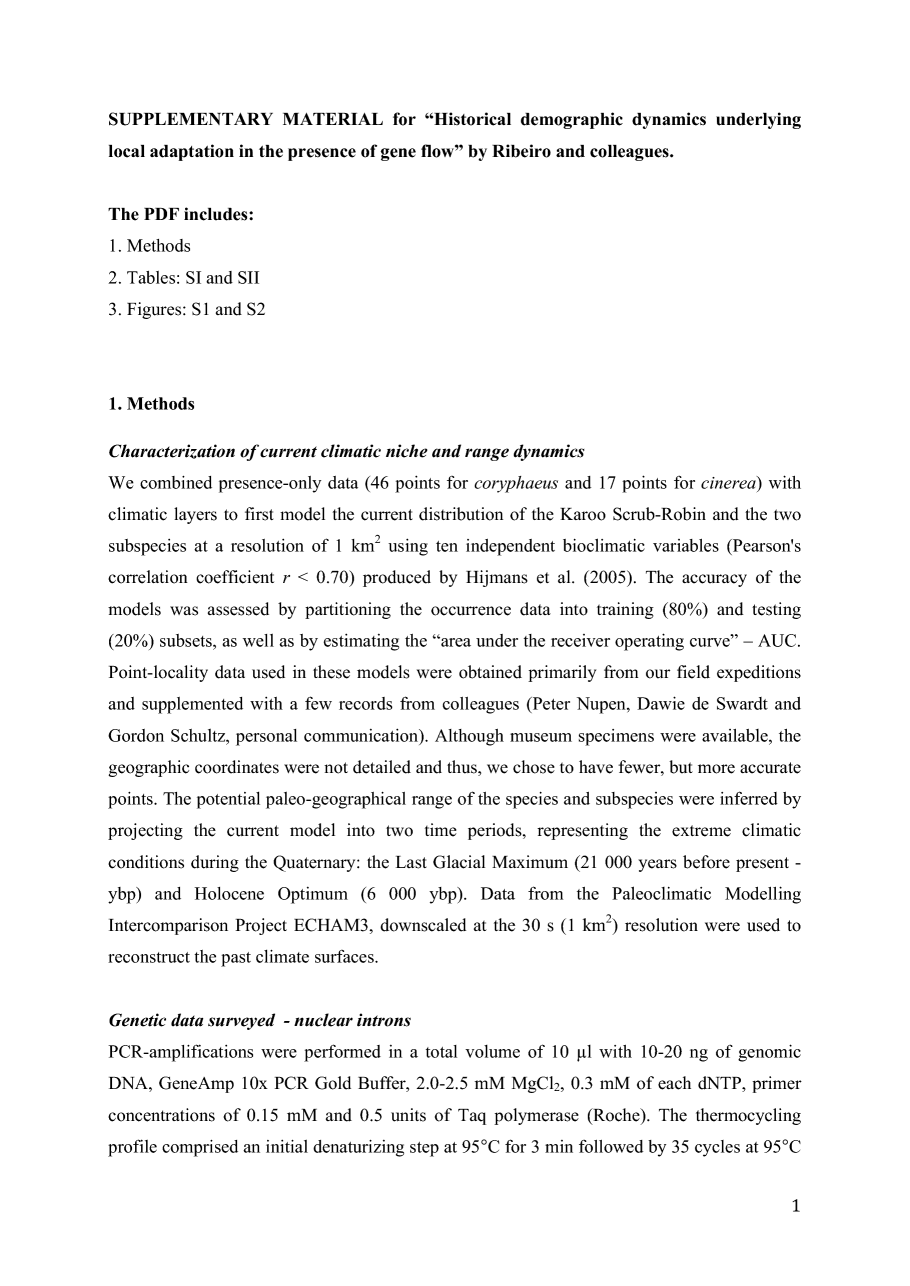

Supplement: Supplementary file 2 [file ece30002-2710-SD2.png]
